# Prevalence and etiology of dentoalveolar trauma in 1,592 United States military working dogs: A 1-year retrospective study

**DOI:** 10.3389/fvets.2022.1102424

**Published:** 2023-01-10

**Authors:** Karin R. Bilyard, Sara B. Mullaney, Travis J. Henry

**Affiliations:** ^1^Midwest Veterinary Dental Services, Elkhorn, WI, United States; ^2^Department of Chemistry and Life Science, United States Military Academy, West Point, NY, United States

**Keywords:** military, working dog, tooth, canine, fracture, dentistry, veterinary

## Abstract

The objective of this study was to quantify the overall prevalence and classification of traumatic dentoalveolar injury (TDI) in a large population of military working dogs (MWDs). The medical records of 1,592 MWDs undergoing routine oral exam and periodontal treatment over a 1-year period were reviewed. The MWDs were located at over 100 military veterinary treatment facilities across the globe. Patient signalment, occupational duty certification, tooth injured, and trauma etiology were recorded. The overall prevalence of TDI was 43.6%. The mean number of TDI per MWD was 1.2. Maxillary tooth fractures were the most common at 60.9% compared to mandibular tooth fractures 39.1%. The most common TDI was enamel-dentin-pulp fractures which accounted for 59.9% of all injuries. Specialized Search Dogs (SSDs) had the highest average of enamel-dentin and enamel-dentin-pulp tooth trauma. Incidental findings with an unknown cause accounted for the majority of tooth trauma 69.2% followed by housing 18.2%, bite work 6.2%, and blunt force trauma 6.0%. The frequency of TDI in the MWD population was substantial, with more than one out of every four MWDs requiring treatment. The probability of a tooth injury in the MWD population was nearly double compared to the pet dog population. Tooth type and age were significant predictors of severe tooth trauma requiring treatment. Improved understanding of MWD tooth trauma prevalence and risk factors will help drive change while maintaining deployment readiness of the team.

## Introduction

Military working dogs (MWDs) are a force multiplier within the Department of Defense (DOD) and serve in a variety of roles throughout the World. They have functioned as warfighters dating back to the mid 1800's (1). Since then, the Army established a “War Dog” program in the Quartermaster Corps in 1942. Military working dogs begin their initial training at the Department of Defense military working dog training program between 12 and 36 months of age. They spend 6–9 months going through training and medical evaluations to ensure they meet the military program requirements. From that point forward, the MWD is housed in a kennel facility and resides on the military installation until they retire from service. During their career, MWDs deploy to a variety of locations but always return to their home duty site. Military working dogs are dual purpose certified meaning that they perform bite work and scent detection.

A working dog primarily uses its oral cavity as a device to perform trained tasks. The oral cavity, teeth, and olfactory system are all anatomically connected through thin alveolar bone, blood vessels, and nerves. Multiple disease processes can concurrently affect both oral and nasal structures. Traumatic dentoalveolar injuries are a collection of specific injuries to the tooth or tooth supporting structures sustained as a direct result of a traumatic force (2). In the MWD, these forces may be caused by abnormal chewing behaviors in the kennel, training rewards, bite work (decoy) training, or blunt force trauma (falling or running into stationary objects).

It has been hypothesized that the working dog populations have a higher prevalence of tooth trauma due to the nature of their work. However, a definitive study of working dog traumatic tooth injury and incidence is long overdue with the most relevant studies dating back to the late 1900's. To the author's knowledge, this is the first paper that performs statistical evaluation on associations, prevalence, and the classification of tooth trauma specific to a working dog population.

The United States Army is the Department of Defense (DOD) lead service for veterinary services and is the only branch of the military that employs veterinarians to provide full-service care to military working dogs. A 2021 military study documented that the second most common condition encountered in MWDs presenting to Army veterinary services in the United States was dental related conditions (3). This study also identified from 2014 to 2015 the most common primary encounter category to receive Army veterinary services for MWDs in Afghanistan was dental, followed by surgical, soft-tissue injury, dermatology, and musculoskeletal. Another military study from 2019 demonstrated the leading conditions affecting 774 young, non-deployed MWDs were: dermatologic (54%), alimentary (46%), dental (34%), soft-tissue-related injury (28%), and musculoskeletal (14%) (4). The primary reasons for inciting dental treatments were tooth extraction, fracture of tooth, root canal, periodontitis, and routine periodontal treatment. In civilian (non-military) working dog populations, a 2013 study reported that police dogs presented to the veterinary emergency services for the following problems: gastrointestinal (28.3%), orthopedic (25.4%), and trauma/wound (15.2%). Dental conditions were listed as occurring in 1.4% of the population (5). In mixed populations of working dogs and pets, tooth fracture prevalence in dogs has been historically reported between 2.6% and 27% (6, 7). A study from 2015 focusing only on the pet population revealed the prevalence of traumatic dentoalveolar injury (TDI) was 26.2% with the mean TDI per patient to be 1.45 (8).

This study identified the prevalence, classification, and associations of TDI in the MWD population. Evaluating the occurrence and risk factors connected to traumatic tooth injuries in MWDs will help stakeholders uncover effective methods for prevention and justification for timely treatment. Ultimately, continued research in this field is critical to understand how oral trauma and/or disease might hinder overall effectiveness and performance of the working dog team.

## Materials and methods

Medical records from January 1, 2019 to December 31, 2019 were obtained from the electronic DOD Veterinary Health Record (VHR), a secure web-based application for world-wide use. The total number of MWDs in active service during the calendar year 2019 was 2,650 dogs. Per military regulations, it is advised that MWDs have an awake oral exam every 6 months and a routine periodontal treatment under anesthesia by an Army veterinarian every year (9). The software was queried for all active MWDs that had routine periodontal treatment performed under anesthesia during the specified timeframe. The total number of MWDs receiving routine periodontal treatment under anesthesia was 1,592. Military working dogs documented as receiving treatment for tooth trauma in 2019 were included in the study. Excluded from the study were MWDs with previous tooth trauma, extractions performed due to periodontal disease, and non-DOD owned working dogs housed off the military base (Transportation Security Administration, Customs, and Border Patrol). Unlike the MWDs, other non-DOD working dogs and civilian police dogs live in home environments with their handlers and have different management programs. The total number of MWDs that were identified as having active traumatic tooth injury during the study period was 1,367.

The MWDs were logged by control number to ensure duplicate patient entries were not created. The injuries were recorded on a tooth-by-tooth basis to allow multiple injuries per patient. The American Veterinary Dental College defines an uncomplicated crown fracture as a fracture of the crown that does not expose the pulp and a complicated crown fracture as a fracture of the crown that exposes pulp (2). In their place, the terms enamel-dentin fracture (when referring to an uncomplicated tooth fracture) and enamel-dentin-pulp fracture (when referring to a complicated tooth fracture) was used in this study. The overall study prevalence of TDI and the total number of MWDs with enamel-dentin-pulp fractures, enamel-dentin fractures, and other TDI injuries (including concussion, enamel fractures, luxation, and avulsion) were calculated and recorded. Abrasion is defined as a pathologic wearing-away of dental hard tissue by the friction of a foreign body independent of occlusion (2). This study did not recognize abrasion as a TDI but it was noted as a common dental finding.

Military working dog demographics, occupational duty certification, age at time of tooth trauma, and tooth fractured were collected for all MWDs with a TDI. All female dogs obtained by the military were spayed at the time of procurement. Male dogs were kept sexually intact unless a medical problem prompted castration. Due to the large population size and provider inconsistencies in the recorded TDI classifications, enamel-dentin-pulp fractures receiving treatment was the only category included in evaluation of tooth trauma etiology. The inciting cause of trauma was classified as: (1) unknown, (2) housing (cage biting and pan chewing), (3) bite work (decoy and sleeve use), and (4) blunt force trauma.

## Statistical methods

Descriptive statistics for categorical variables (sex, breed, certification, patrol function, tooth type, and age category) included frequency and percentages. Age was described by histogram, median, and range.

Age category variable was manually created by assigning age ranges into four categories. Category 1: <24 months, category 2: 24 to <48 months, category 3: 48 to <72 months, and category 4: ≥72 months. The patrol variable was manually created by assigning all dogs not certified in patrol to one category and assigning all dogs certified in patrol to a separate category. Differences in proportion were assessed by Pearson's chi-square test.

Univariable and multivariable logistic regression models were used to identify factors associated with the occurrence of enamel-dentin-pulp fractures. The outcome variable for the analysis was having an enamel-dentin-pulp fracture and the independent variables were the demographic characteristics of the military working dogs including sex, breed, certification, patrol, tooth category, and age category. In the multivariable analysis, the full model included all variables with *P*-value <0.25 from the univariable analysis. Variables were removed stepwise, starting with the highest *P*-value until all variables with *P*-value >0.05 were removed. Independent variables were assessed for confounding by looking for a change in model coefficients of ≥10% as variables were removed from/added to the model. The Pearson goodness-of-fit test and the Hosmer and Lemeshow Goodness of Fit Test *P*-values indicated good model fit.

Descriptive statistics were performed using Excel Office 365 version 2205. Statistical analyses were performed using SAS^®^ Studio Release 3.8 (SAS Institute Inc., Cary, NC, USA), and StatCrunch (Pearson Education Inc., 2019). The statistical significance level for these results was <0.05.

## Results

**Figure 1:**
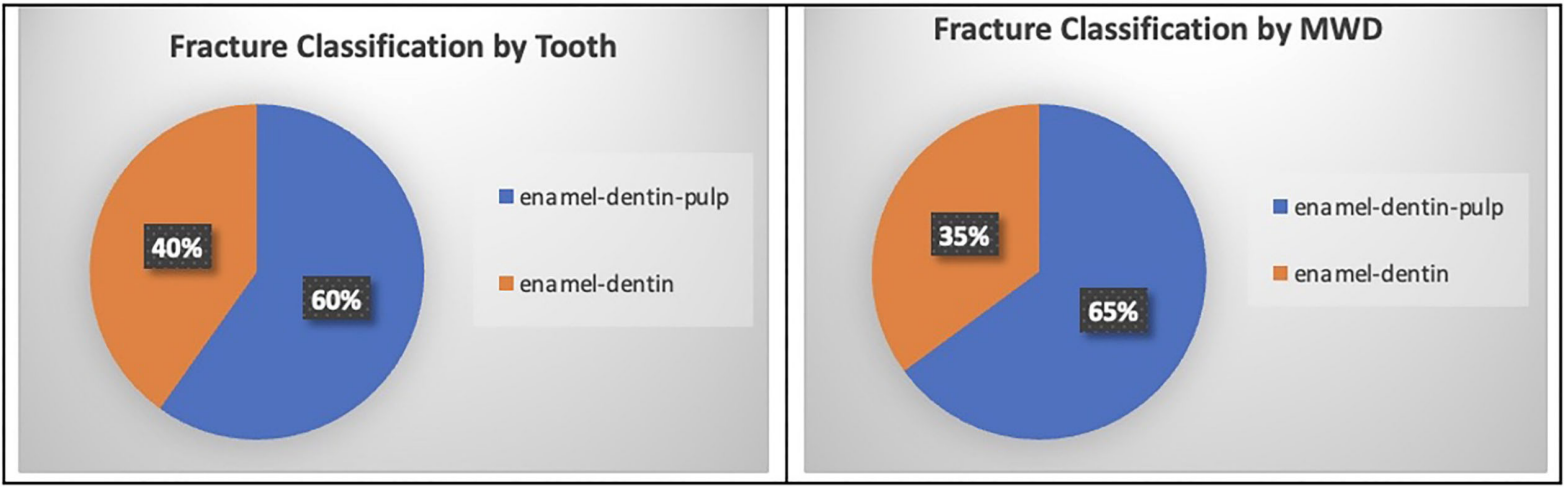
Fracture classification (enamel-dentin-pulp and enamel-dentin) percent by total tooth count and by total MWD population count.

Overall, 1,592 MWDs were anesthetized for routine oral exam and periodontal treatment. Two hundred and twenty-five MWDs were excluded for not meeting the study criteria. The study consisted of 1,367 active United States MWDs stationed worldwide at over 100 identified military installations. A total of 596 MWDs had TDIs identified with a total of 1,081 teeth affected. The prevalence of at least one TDI was 43.6%. The mean total TDI injuries per patient was 1.2. The majority of the TDIs identified could not be classified beyond enamel-dentin fractures (434 [40.1%]) and enamel-dentin-pulp fractures (647 [59.9%]). Military working dogs that fractured a tooth exhibiting enamel-dentin-pulp exposure totaled (387 [64.9%]) which averages to 1.1 teeth per MWD. Military working dogs that fractured a tooth exhibiting enamel-dentin exposure totaled (209 [35.1%]) which averages to 1.4 teeth per MWD (Figure 1).

Male patients were overrepresented (456 [76.5%]) compared to female patients (140 [23.5%]). This included males (73 [12.2%] castrated and 383 [64.3%] sexually intact) and females (140 [23.5%] spayed). There was no statistical significance between sex and enamel-dentin-pulp fractures (*P* = 0.82).

A total of six dog breeds were included in the study population. The most common dog breeds in the study were Belgian Malinois (316 [53%]) and German Shepherds (225 [37.8%]). The other breeds included were Dutch Shepherds (18 [3%]), Labrador Retrievers (31 [5.2%]), German Shorthair Pointers (4 [0.7%]), and other (2 [0.3%]). Breed played no role (*P* = 0.84) in risk associated with enamel-dentin-pulp fractures.

**Figure 2:**
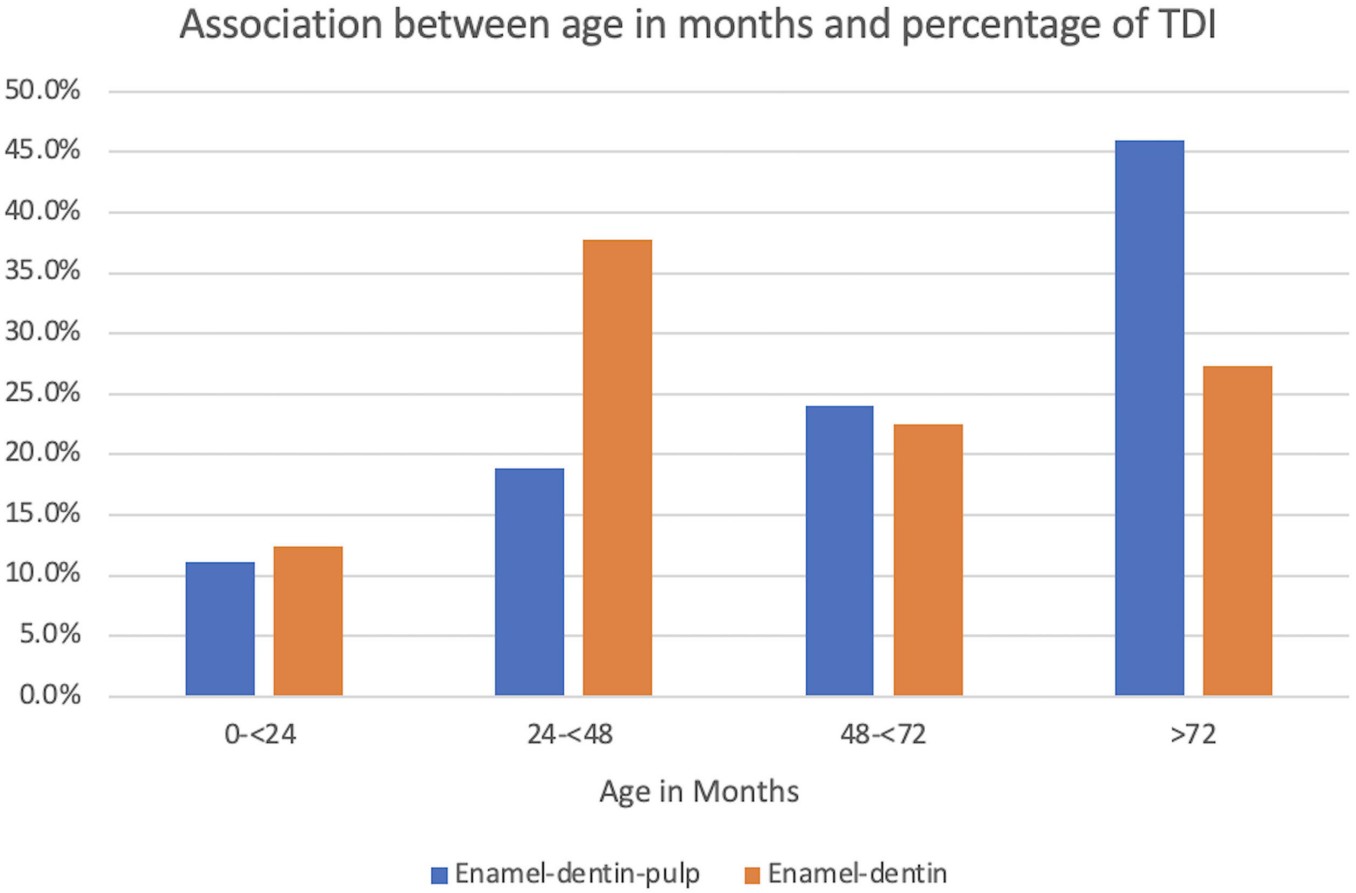
Graphical projection of the association between age in months and the percentage of TDI. Note that the enamel-dentin fractures were highest in the 24–48-month MWDs and the enamel-dentin-pulp fractures were highest in the 72 month and older MWDs. There was statistical significance between enamel-dentin-pulp fractures and age (*P* ≤ 0.0001).

The age of the patients ranged from 14 to 132 months (mean ± SD, 67 ± 30 months; median 69.6 months). The most common age group affected with TDI were the 72 months and older MWDs (235 [39.4%]). The most common age groups affected with enamel-dentin fractures were the 24–48 months MWDs (79 [37.8%]) and the most common age group with enamel-dentin-pulp fractures were the 72 months and older MWDs (178 [46.0%]). The association between enamel-dentin-pulp fractures and age was found to be significant (*P* ≤ 0.0001). Furthermore, when controlling for patrol and risk of enamel-dentin-pulp fractures, MWDs over 72 months of age were 1.98 times more likely to have a fracture with pulp exposure compared to MWDs <24 months of age (Figure 2).

Traumatic dentoalveolar injuries occurred frequently in the incisor teeth (592 [54.8%]) and canine teeth (346 [32.0%]). The premolar teeth and molar teeth accounted for (97 [9.0%]) and (46 [4.3%]), respectively. Most of the injuries occurred in the maxilla (658 [60.9%]). The right maxillary quadrant sustained the most tooth injury (362 [33.5%]) followed in decreasing order with the left maxillary quadrant (296 [27.4%]), the right mandibular quadrant (227 [21.0%]), and the left mandibular quadrant (196 [18.1%]). The teeth commonly injured for all TDI classifications in decreasing order were: the right maxillary canine tooth, the left maxillary canine tooth, the right maxillary second incisor tooth, and the left mandibular canine tooth.

**Figure 3:**
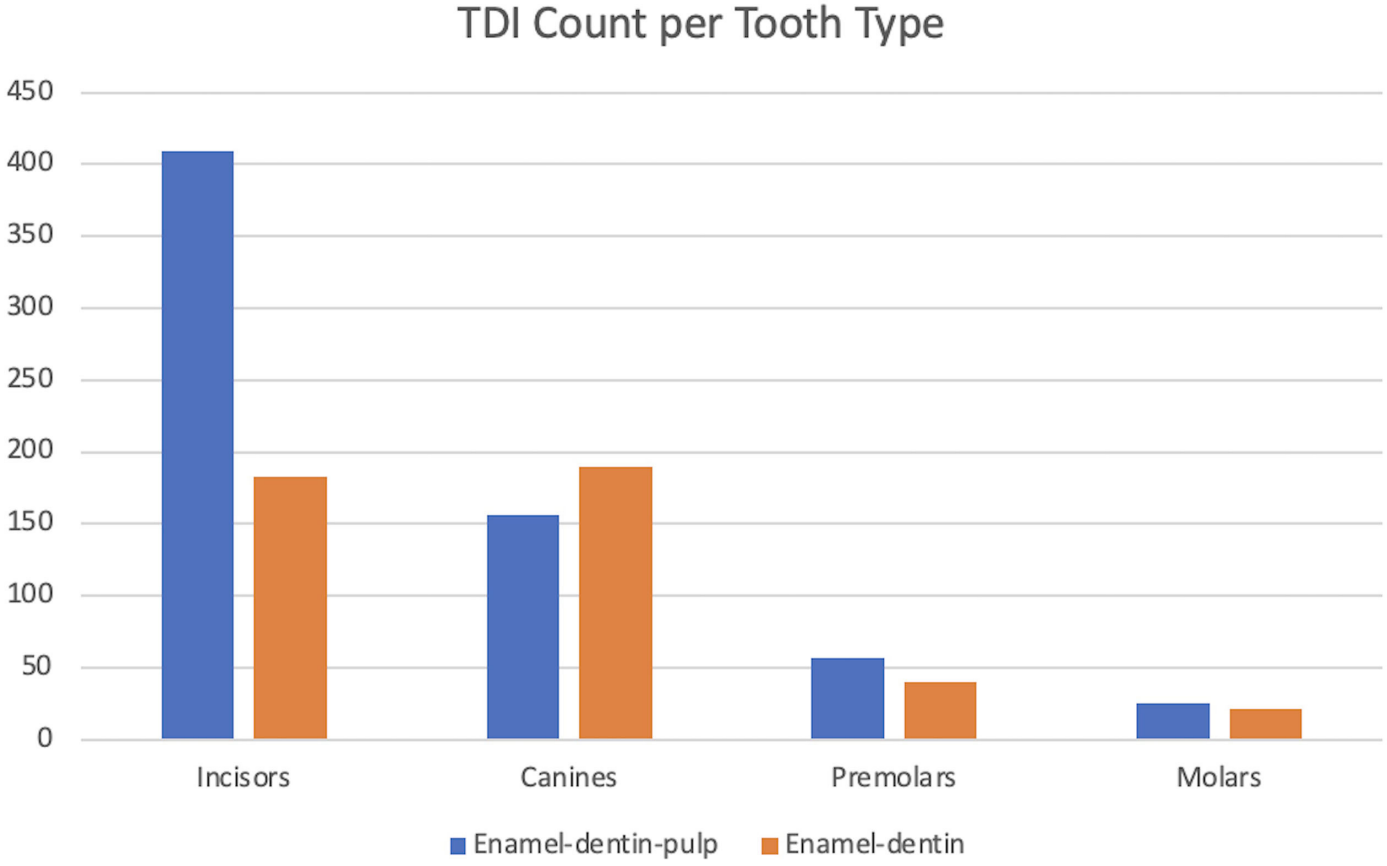
Graphical projection of TDI count and the tooth type. Note that the incisor teeth had the highest incidence of enamel-dentin-pulp fractures, and the canine teeth had the highest incidence of enamel-dentin fractures. The incisor and canine teeth had a significant risk of enamel-dentin-pulp fractures (*P* = 0.008).

**Figure 4:**
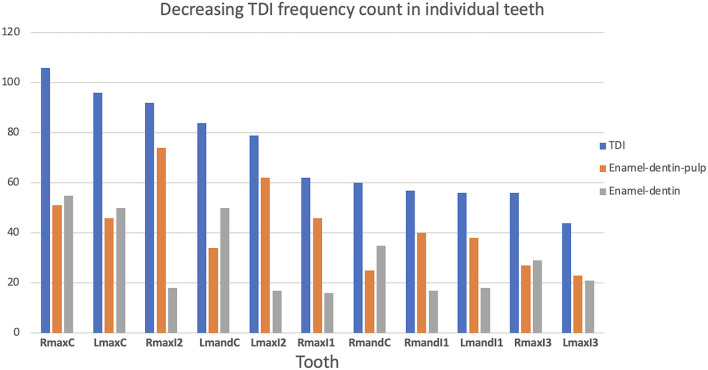
Graphical description of the decreasing frequency of TDI and the occurrence of enamel-dentin-pulp fractures and enamel-dentin fractures in individual teeth.

**Figure 5:**
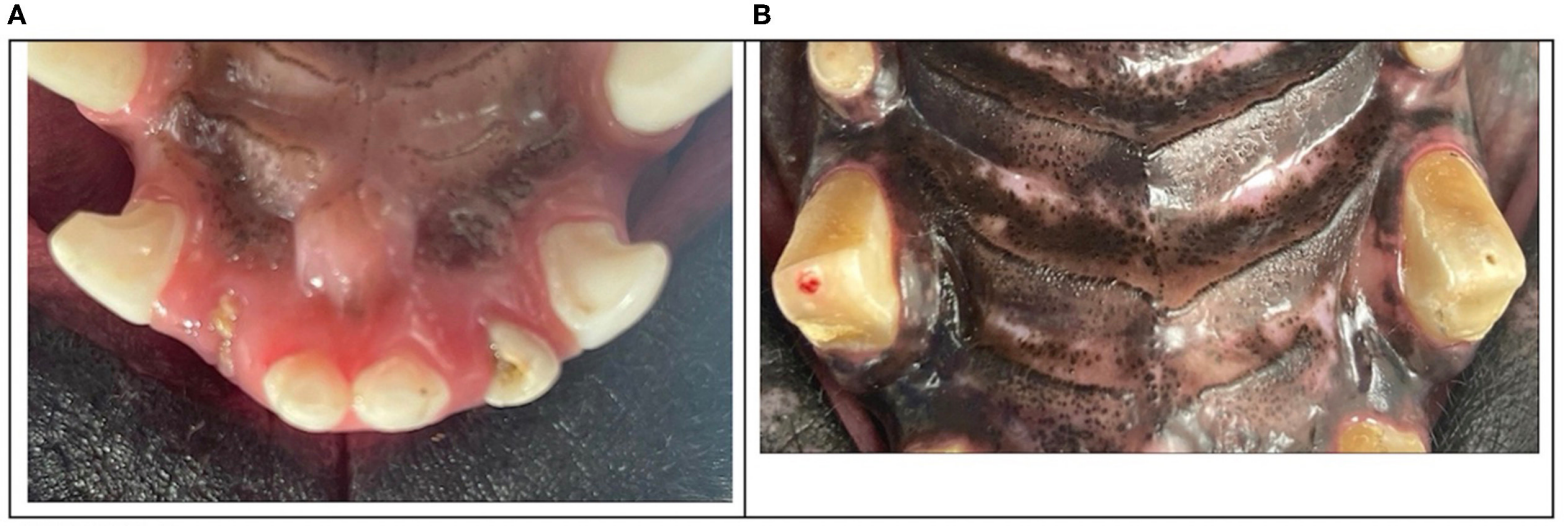
Clinical photographs of the most injured teeth with enamel-dentin-pulp fractures were the right maxillary second incisor tooth, the left maxillary second incisor tooth **(A)**, the right maxillary canine tooth, and the left maxillary canine tooth **(B)**.

In this study, enamel-dentin fractures accounted for 40.1% of all TDIs. The teeth most affected by enamel-dentin fractures were the canine teeth (29.4%) and the incisor teeth (28.3%). Teeth injured with enamel-dentin fractures in decreasing order were: the right maxillary canine tooth, the left maxillary canine tooth equally with the left mandibular canine tooth, the right mandibular canine tooth, and the right maxillary third incisor tooth. This study documented enamel-dentin-pulp fractures accounted for 59.9% of all TDIs. Inversely, the teeth most affected by enamel-dentin-pulp fractures were the incisor teeth (63.2%) and the canine teeth (24.1%). The most injured teeth with enamel-dentin-pulp fractures in decreasing order were: the right maxillary second incisor tooth, the left maxillary second incisor tooth, the right maxillary canine tooth, and the left maxillary canine tooth equally with the right first incisor tooth. The incisor and canine teeth were significantly associated with an increased risk of enamel-dentin-pulp fracture (*P* = 0.008) (Figures 3–5).

**Figure 6:**
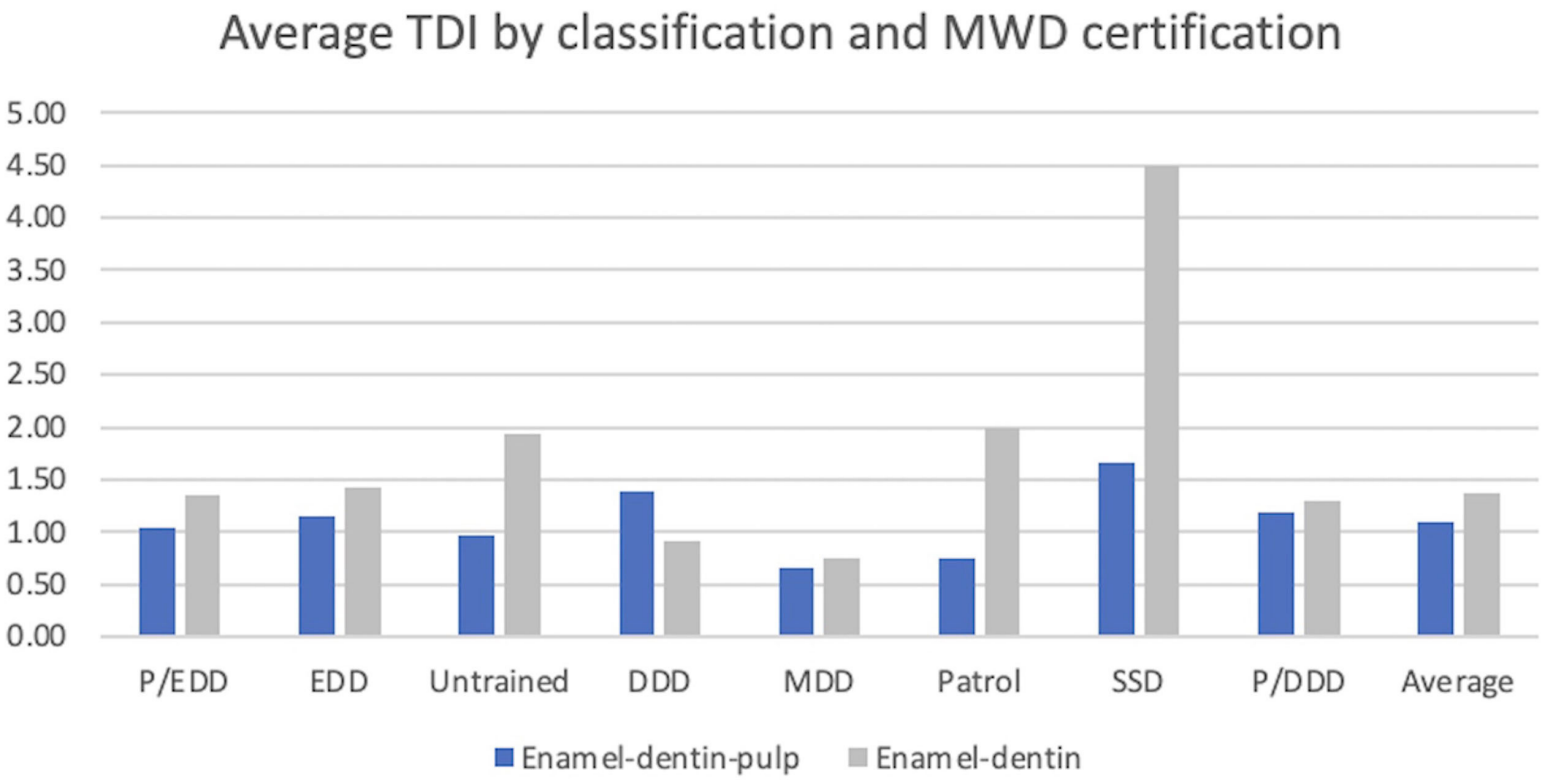
Graphical description of TDI and MWD certification. P/EDD (Patrol/Explosive Detection Dog), EDD (Explosive Detection Dog), Untrained (not certified), DDD (Drug Detection Dog), MDD (Mine Detection Dog), Patrol, SSD (Specialized Search Dog), P/DDD (Patrol/Drug Detection Dog). There was no statistical significance between MWD certification and enamel-dentin-pulp fractures.

The occupational duty certification at time of tooth fracture was recorded. The majority of MWDs are dual purpose certified in both Patrol and Detection work either explosives (58.4%) or Drug Detection (10.9%). Other categories include single purpose MWDs performing: Explosive Detection (18.6%), Drug Detection (3.5%), Mine Detection (0.5%), Specialized Search Detection (1.5%), Patrol only (1.3%), and Untrained (5.2%). The average number of TDI by all certifications combined was 1.2 teeth per MWD. The average number of enamel-dentin-pulp fractures was 1.1 teeth per MWD. Average number of enamel-dentin fractures was 1.4 teeth per MWD. Specialized Search Dogs (SSDs) and Drug Detection Dogs (DDDs) had the highest average of teeth fractured with enamel-dentin-pulp fractures with 1.7 and 1.4, respectively. Specialized Search Dogs and single purpose Patrol had the highest average enamel-dentin tooth fractures with 4.5 and 2.0, respectively (Figure 6).

**Figure 7:**
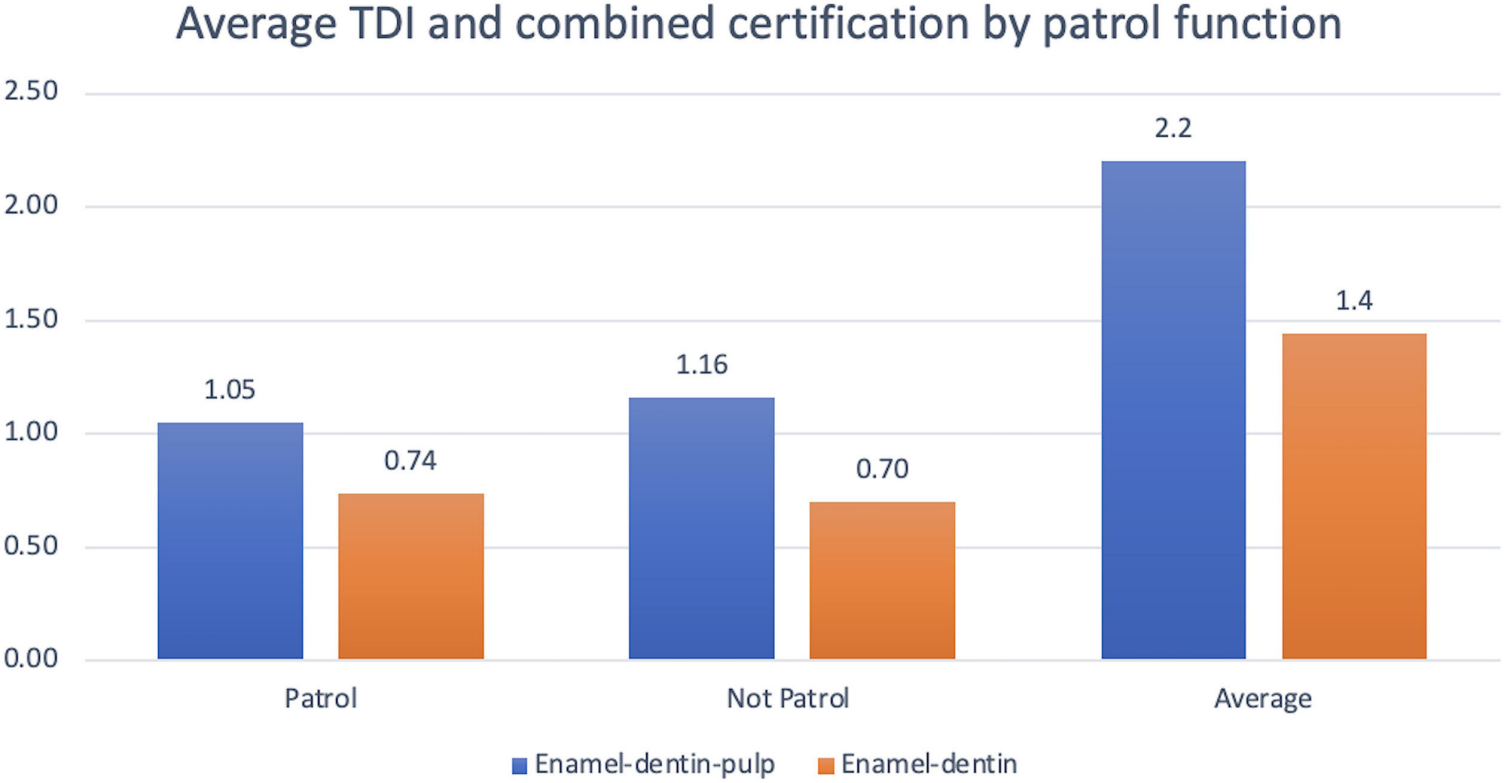
Graphical projection of average TDI and combined certification by patrol function. Note that both patrol and non-patrol MWDs had a higher frequency of enamel-dentin-pulp fractures vs. enamel-dentin fractures. There was no statistical significance between patrol function and increased risk of enamel-dentin-pulp fractures.

There were 421 MWDs performing patrol work and 175 MWDs not performing patrol work. Military working dogs performing patrol work have a 69.8% chance of a TDI and a 41% chance of an enamel-dentin-pulp fracture. Military working dogs not performing patrol work have a 30.2% chance of a TDI and a 18.8% chance of an enamel-dentin-pulp fracture. Neither certification category (*P* = 0.6) nor patrol work (*P* = 0.08) demonstrated a significant association for enamel-dentin-pulp fractures within the MWD population (Figure 7).

**Figure 8:**
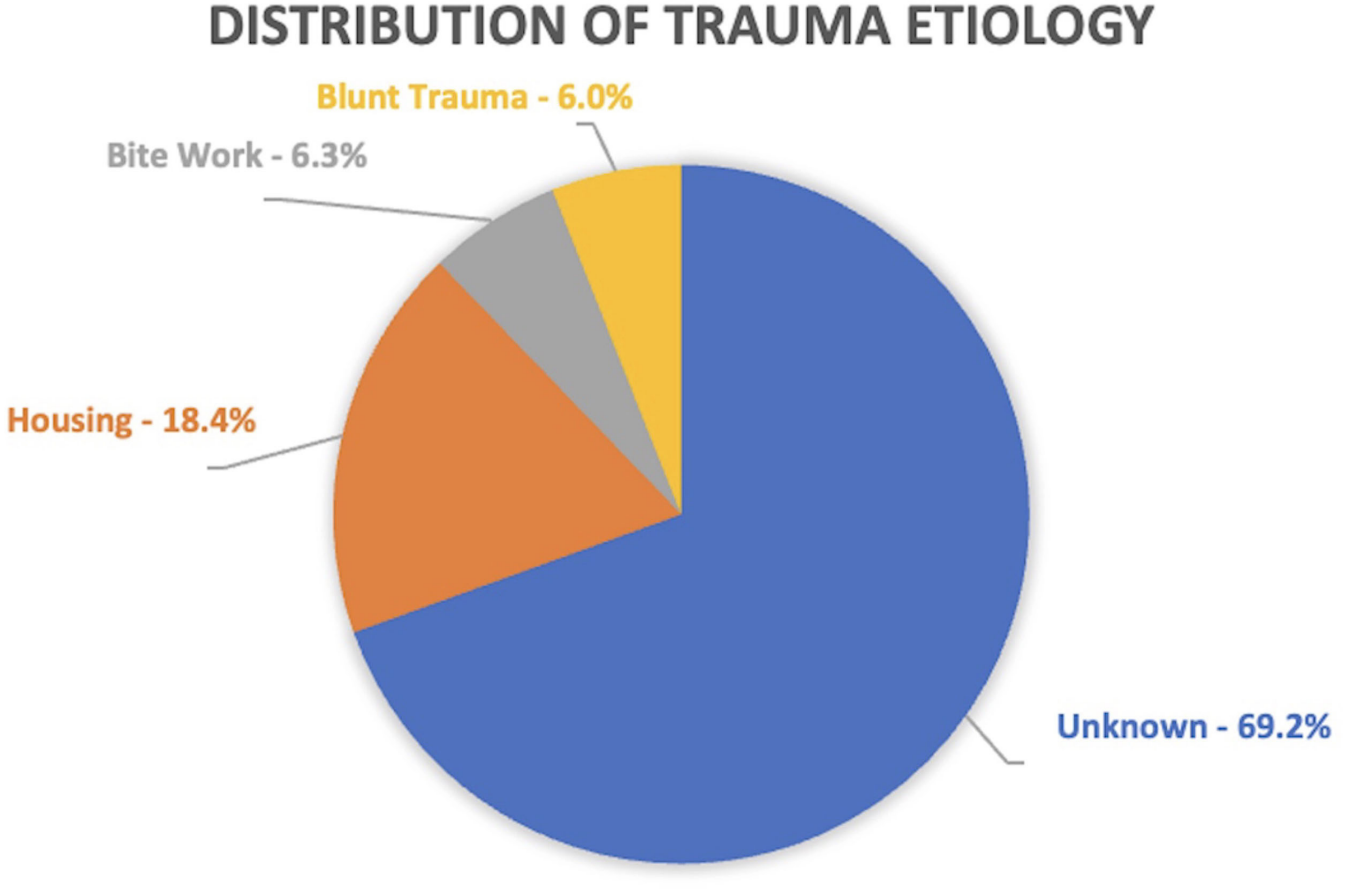
Distribution of trauma etiology. Note that unknown cause accounted for the largest proportion of trauma etiologies.

The distribution of tooth trauma etiology was relatively consistent among the different certifications. The main cause of TDI resulting in enamel-dentin-pulp fracture was unknown and an incidental finding in (448 teeth [69.2%]). The remaining were caused by housing (119 [18.4%]), bite work (41 [6.3%]), and blunt force trauma (39 [6.0%]). The patrol MWDs had a tooth trauma distribution of unknown (296 [70.3%]), housing (83 [19.7%]), bite work (28 [6.7%]), and blunt force trauma (14 [3.3%]). The MWDs not performing patrol work had a trauma distribution of unknown (115 [65.7%]), housing (31 [17.7%]), blunt force trauma (21 [12.0%]), and bite work (8 [4.6%]) (Figure 8).

## Discussion

This study evaluated the prevalence of MWD tooth trauma and details the relationships between job certification, location of injuries, and etiology. The frequency of TDI in the MWD population is nearly double the frequency of TDI in pet populations. Military studies have documented the frequency of dental encounters compared to overall patient encounters but never enumerated the diagnosis of each encounter within the study sample (3, 4, 10). Results from pet population studies quantify TDI frequency anywhere between 14% and 27% with the most recent study reporting 26.2% (7, 8). A study of canine teeth fractured in French military dogs demonstrated that 25% of the dogs had at least one canine tooth fractured (10). The data from this study for frequency of TDI in the United States MWD population is 43.6%. Based on the combined data, it is evident that the military working dog population fractures their teeth at a higher frequency compared to pet populations. Specifically, MWDs are nearly twice as likely to experience tooth trauma.

**Figure 9:**
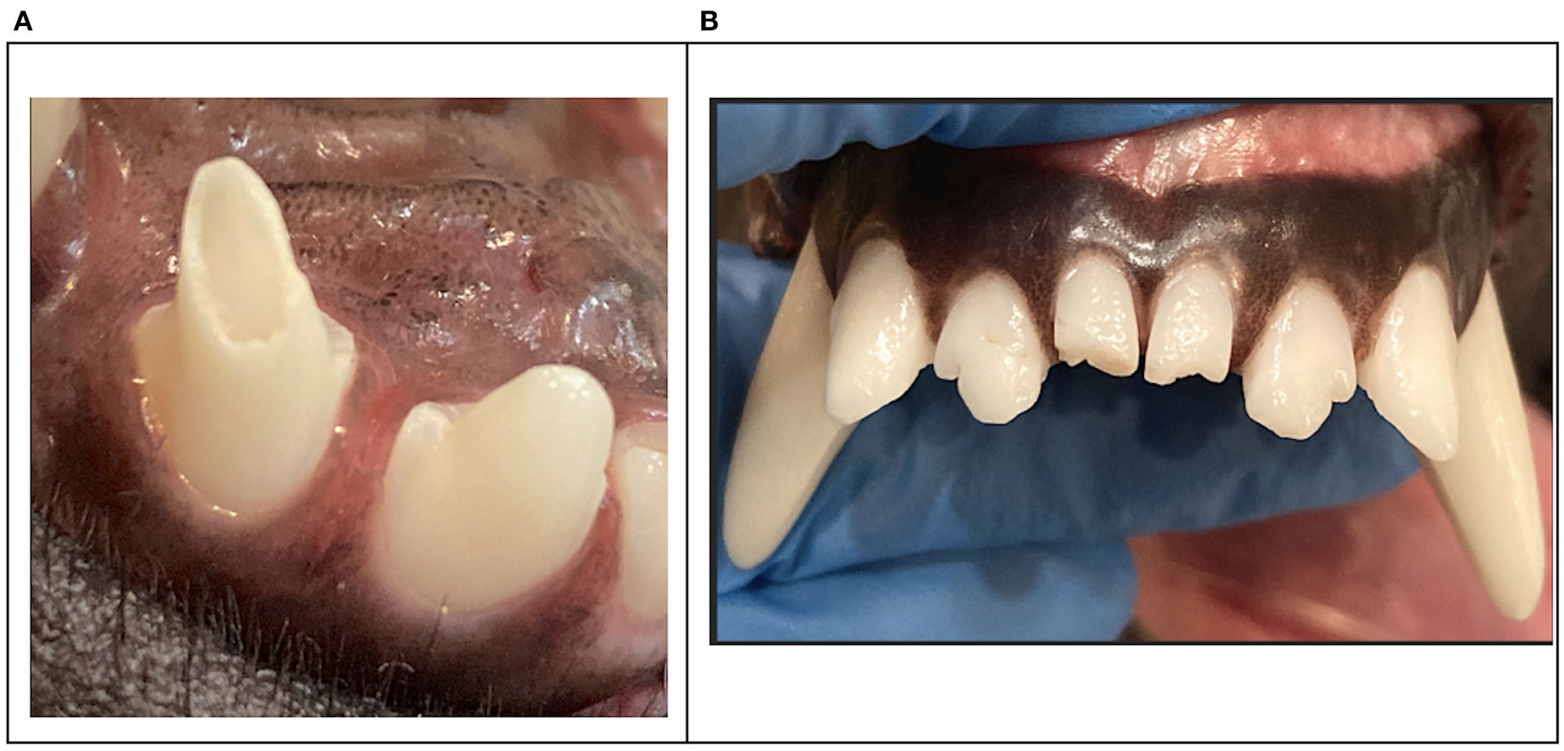
Clinical photographs demonstrating an enamel-dentin fracture on the maxillary third incisor **(A)** and enamel-dentin fracture on the right and left mandibular first incisors **(B)**.

Tooth type and location within the oral cavity is a significant predictor for enamel-dentin-pulp fractures. When all MWD fracture injuries were combined, the incisor teeth had the highest frequency (54.8%) followed by the canine teeth (32.0%), the premolar teeth (9.0%), and the molar teeth (4.3%). The probability of fracturing an incisor tooth was 1.5 times more likely compared to a canine tooth. Capík et al. reported mandibular canine teeth, maxillary premolar teeth, and maxillary incisor teeth were the most common teeth fractured in pet dogs (6). In 2015, Soukup et al. combined all fracture injuries and reported the premolar teeth had the highest frequency (39.0%), followed by the canine teeth (33.3%), the incisor teeth (21.0%), and the molar teeth (6.7%) (8). Premolar tooth fractures are common in pets but rarely seen in MWDs. Our findings in this study only identified 14 maxillary fourth premolar tooth fractures accounting for (0.2%) of total tooth injury. Pet dogs' fracture maxillary premolars (specifically the right and left maxillary fourth premolar teeth) during playful chewing on hard objects. Military working dogs do not receive objects that are hard, but their kennel design, daily management, training protocols, and occupational duties enhance the risk for trauma of the rostral dentition (Figure 9).

Most injuries occurred in the maxilla (60.9%) and the right quadrants of both the maxilla and mandible sustained the most tooth trauma compared to the left. One theory for the pattern of injury sustained to the right tooth quadrants is that during initial training the MWDs are only trained on soft to hard arm sleeves with a hard plastic elbow protector. Most master trainers and decoys wear the sleeve on their left arm placing the hardest part of the sleeve on the MWDs right side of occlusion. This protocol for training is conducted when the MWD is between 12 and 36 months of age.

Freeman et al. found that the cumulative effects of lower dentin strength and a larger pulp cavity reduce the strength of the base of the juvenile tooth by almost half (48%) (11). Consequently, standard operating procedures for young MWDs should incorporate awareness of the stress forces on teeth that occur during bite training and tugging/pulling interactions with high value rewards. Once the MWD is transported to his permanent military location, the training protocol incorporates full bite suits, jackets, and sleeves to advance the MWDs apprehension capabilities.

**Figure 10:**
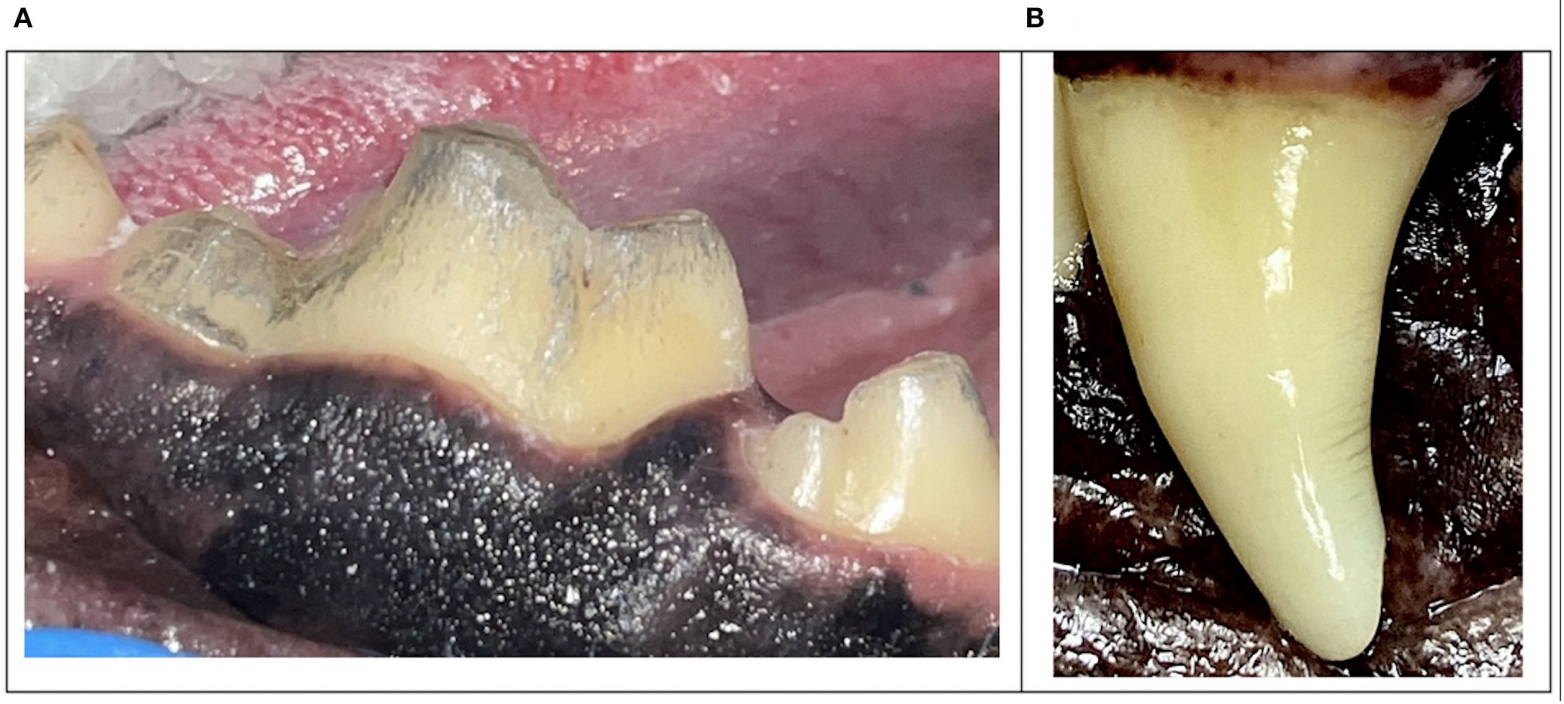
Clinical photos of extrinsic metallic staining **(A)** and enamel infractions **(B)**.

Tooth fractures requiring treatment is inevitable as 59.9% of the total teeth injured resulted in pulp exposure. Enamel-dentin-pulp fractures in decreasing order occurred in the right maxillary second incisor tooth, the left maxillary second incisor tooth, the right maxillary canine tooth, the left maxillary canine tooth, and the right maxillary first incisor tooth being equal. Enamel-dentin-pulp fractures were also the most common TDI (49.6%) in pet populations and involved the premolar and canine teeth (8). This author has observed that MWD canine tooth fractures often present with oblique crown fractures in a distal-mesial fracture pattern. A study on canine tooth fractures in French military dogs observed that most canine teeth, when fractured, resulted in pulp exposure with more that 50% of the clinical crown lost. This same study also documented that military dogs fracture their canine teeth in an oblique, distal-mesial pattern but also noted horizontal splits on the distal surface of the crowns (10). Working dogs may present with enamel infractions or horizontal “craze” lines on the distal surface of their canine teeth. The direction of crack propagation is generally perpendicular to the stress direction and perpendicular to the surface (12). These cracks are subtle but can be an early indicator of inappropriate chewing behavior or previous tooth trauma. Lawn et al. determined that transverse fractures of the canine teeth form first as channel cracks in the enamel, arrest at the dentin–enamel interface, and then penetrate the dentin before becoming unstable (13). In 2017, Goldschmidt et al. noted a trend to see more enamel-dentin fractures at the distal ridge of canine teeth during distal-mesial force direction fracture loading (14). Extrinsic staining on teeth may also be an early clinical exam finding that proposes impending catastrophic tooth trauma. The clinician should be aware of these abnormal findings so that early intervention and management might help reduce additional tooth trauma (Figure 10).

Surprisingly, this study did not support our hypothesis that patrol MWDs have a higher incidence of tooth trauma based on the nature of their work. There was no statistical significance between certification category and greater incidence of TDI with patrol functions. Although bite suit/sleeve materials and apprehension training methods do cause tooth trauma, it was the third most reported cause of TDIs in MWDs. Unexpectedly, non-patrol MWDs such as Specialized Search Dogs (SSDs) and Drug Detection Dogs (DDDs) had the highest average teeth fractured when compared to MWDs performing patrol work. One thought is that the duties of SSD and DDD dogs involve tracking odor with their noses close to the ground while moving in obscure patterns. Many SSDs are employed off-leash while searching for buried objects. This work environment might increase their risk of blunt tooth trauma on stationary objects. Another consideration specific to the United States MWD program is that many branches within the DOD are phasing out the single purpose MWDs and prefer a multi-purpose or dual purpose MWD, thus, there were fewer single purpose MWDs in this study. Moreover, the lack of relationship between certification and tooth trauma must be interpreted with caution as some dual purpose MWDs transition to single purpose MWDs during their career. Our study documented certification at time of tooth trauma identification, so it is likely that the tooth was injured as a result of patrol work prior to identification.

Age was found to be a significant predictor for TDI in the MWD population. Military working dogs over the age of 72 months were almost twice as likely to suffer a TDI with pulp exposure when compared to younger patients. The median age of combined TDI was 68 months. Military working dogs over 72 months of age had the highest risk of enamel-dentin-pulp fractures at 46%. As the MWD ages, the probability of enamel-dentin-pulp fracture increases and nearly doubles from 48 to 72 months. Conversely, MWDs between 24 and 48 months of age had the highest probability of enamel-dentin fractures at 37.8%. This age group is twice as likely to encounter an enamel-dentin fracture compared to an enamel-dentin-pulp fracture. Military working dogs with enamel-dentin fractures have a weakened tooth structure which increases the risk of impending catastrophic fracture later in life. The increased probability of enamel-dentin-pulp fractures as the MWD ages supports this hypothesis. Dentin thickness, radius of canal curvature, and external root morphology all interact in influencing fracture susceptibility and the pattern of fracture (12). Familiarity and knowledge on the vulnerable tooth structure in young MWDs could proactively minimize enamel-dentin fractures attained early in the MWDs career.

Generalized abrasion was not included in the study, however, it is a common pathology noted by clinicians during periodontal treatment. Abrasion is an irreversible process that changes tooth shape, affects function, and compromises structural integrity. Military working dog abrasion patterns normally exhibit occlusal surface abrasion on the incisor teeth, premolar teeth, and molar teeth, and the canine teeth exhibit distal surface abrasion.

**Figure 11:**
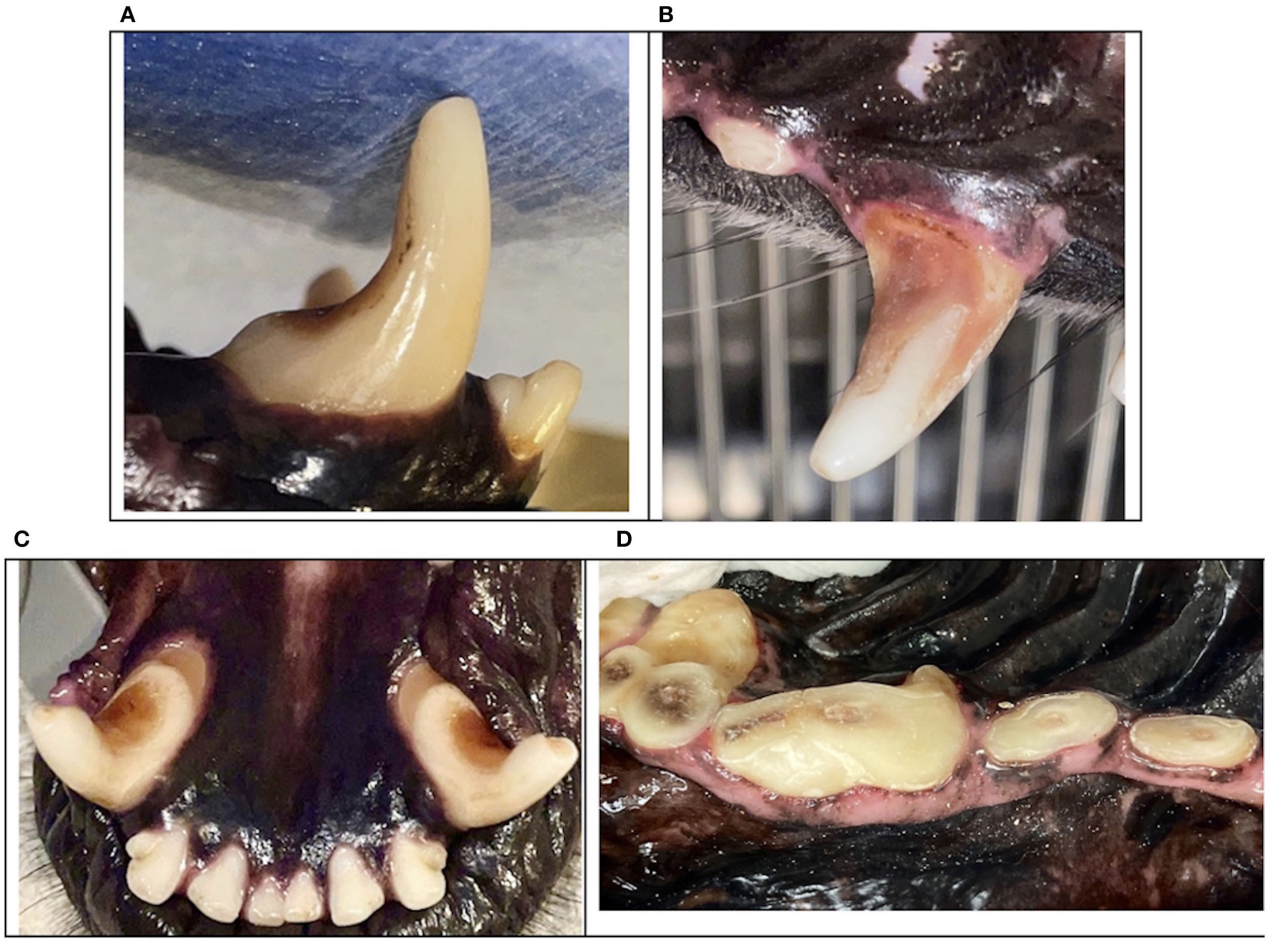
Clinical photographs demonstrating severe distal abrasion on canine teeth **(A–C)** and occlusal abrasion on maxillary pre-molar and molar teeth **(D)**.

**Figure 12:**
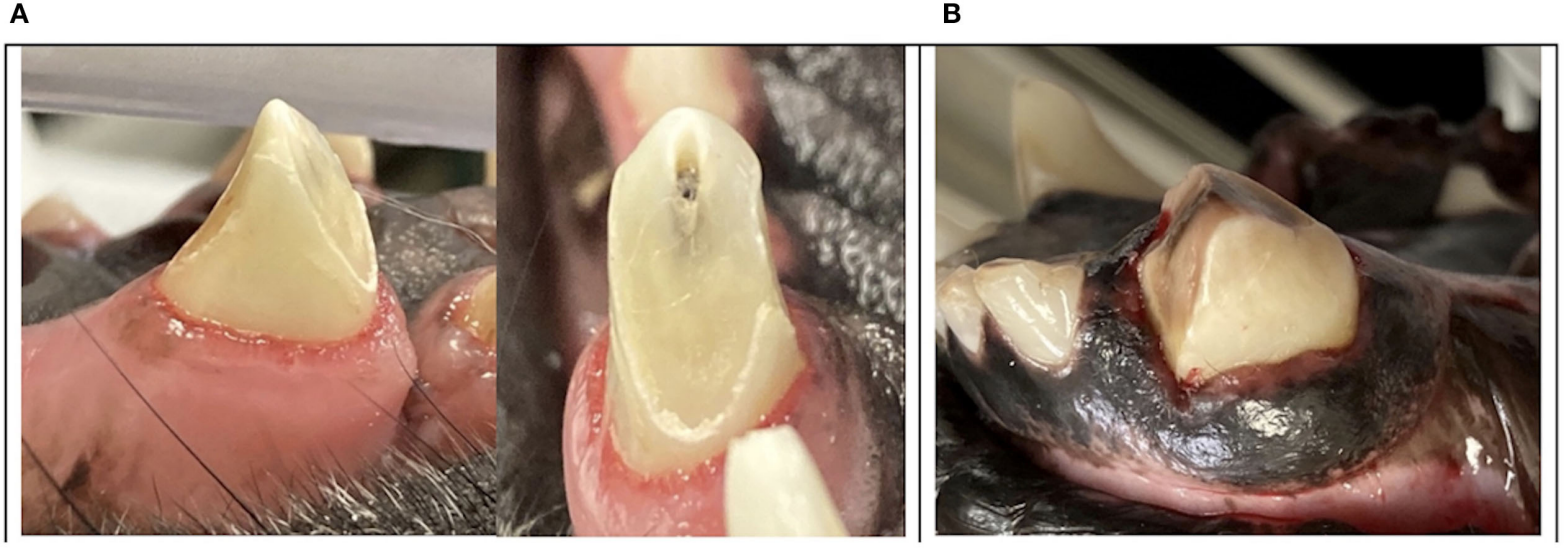
Clinical photographs of trauma to the distal surface of canine teeth leading to catastrophic oblique crown fractures with propagation in the distal-mesial direction **(A, B)**.

Goodman et al. demonstrated that nearly 25% of maxillary fourth premolar teeth with enamel-dentin fractures had radiographic evidence of endodontic disease (15). A study of French military dogs reported that 85.7% of dogs with a fractured canine tooth had abrasion noted on the distal surface of the remaining canine teeth (10). The more dentin is removed, the greater the fracture susceptibility (12). These studies confirm that endodontic disease with abrasion or enamel-dentin fractures are likely to require treatment. Similarly, the depth and pattern of abrasion increases the probability of tooth fracture. Differentiating between enamel-dentin fractures and abrasion are important as the underlying etiology and treatment are often different (Figures 11, 12).

**Figure 13:**
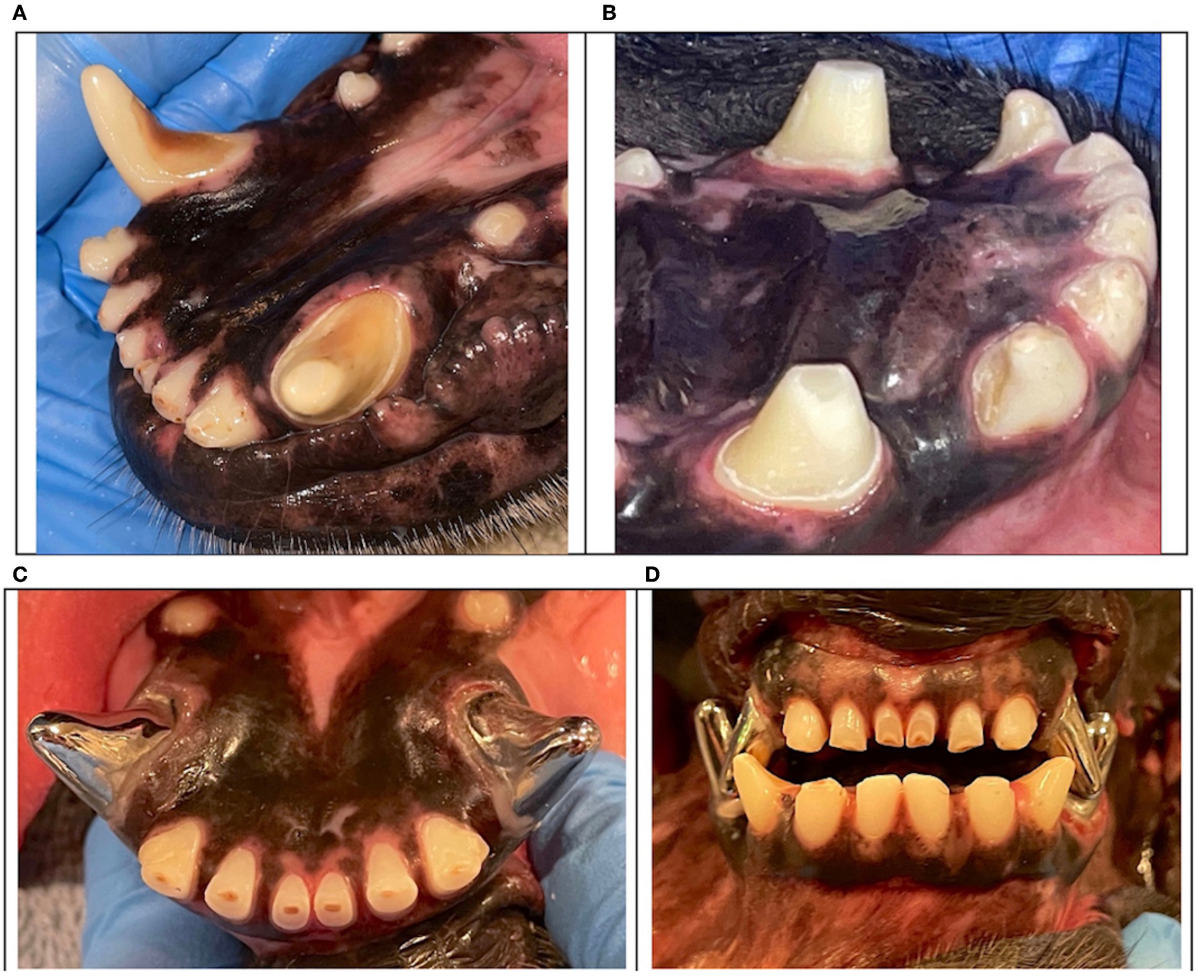
Clinical photographs demonstrating significant trauma to the distal surface of the lower mandibular canines **(A)** with crown shortening, endodontic therapy, and prosthodontic crown preparation **(B)**. Crown delivery of the lower mandibular canines **(C)** and crown delivery of both the maxillary and mandibular canines in a young MWD to preserve a functional occlusion **(D)**.

This data brings into question whether prevention and early intervention of enamel-dentin fractures and severe abrasion could prevent further tooth trauma. If proactive measures are instituted to reduce tooth trauma by addressing management and training protocols, there may be a reduction of catastrophic tooth fractures in the MWD population. Subsequently, treatments designed to safeguard the abraded tooth from catastrophic fracture such as crown reduction with endodontic procedures and prosthodontic crown placement may prove beneficial in these patients (Figure 13).

The nature of all TDI types were associated with unknown cause, housing, bite work, and blunt force trauma. Unknown cause of tooth trauma occurring in 69% of the MWD population is consistent with previous studies of pet population trauma etiology. The cause and timing of injury is often described as “unknown” as stated by the handler. Apparent physical symptoms of oral trauma are not commonly observed. Most history documented in the medical records indicate a training deficiency or change in behavior noted by trainers and handlers. The most common physical observations are a MWDs reluctance to bite the target, a shallow bite, early release of the bite, “chattering” on the sleeve, or difficulty holding with a deep bite. Efforts should be made to document detailed information about the fracture cause and pattern to improve data analysis. Handlers should be trained on identifying oral trauma and performing daily oral exams. In doing so, veterinary providers can monitor TDI prevalence and intervene with preventive care prior to catastrophic injury. Furthermore, incidental trauma recognized during routine periodontal treatment was a common finding, thus emphasizes the importance of annual oral exams under anesthesia.

The second most common factor influencing tooth trauma is the housing environment for both patrol MWDs and non-patrol MWDs. The majority of MWD kennels have residential style chain link fencing for housing perimeters. It's likely that MWDs injure their maxillary second incisors and canine teeth by the pulling/tugging forces applied during inappropriate chewing behaviors while in confinement. This would also explain the oblique fracture patterns seen on most canine teeth of patrol and non-patrol MWDs. Goldschmidt et al. was able to document when forces are applied in the distal-mesial direction, an oblique crown fracture pattern occurs in 85.7% of the population (14). A study on fence mesh size compared to anatomical spacing of teeth might confirm tooth trauma consistent with cage biting/pulling. Additionally, a comparative study evaluating the prevalence of tooth fractures between military dogs housed in kennels on military installations verses non-military working dogs housed in home environments with their handlers could further identify potential risks with kennel design, training programs, exercise requirements, and management.

Military working dogs have robust capabilities making them a highly effective team asset. Nearly half of the MWD study population experienced some type of a TDI with 30% of injuries requiring treatment due to traumatic pulp exposure. The frequency of TDI in the military working dog population is significantly more than the pet population. Catastrophic tooth fractures requiring treatment should be expected by any veterinarian providing services to MWDs. Clinical management of TDI should focus on saving strategic teeth (the mandibular first molar teeth, the mandibular and maxillary canine teeth, and the maxillary fourth premolar teeth). Maintaining the function of these teeth with advanced dental procedures allows preservation of bite forces and sustains occlusal biomechanics. Performing appropriate endodontic and prosthodontic procedures permits a faster recovery and strengthens operational effectiveness by minimizing down time of the MWD team.

The information available from this study can serve as a baseline for further in-depth analyses of dental trauma mitigation, improved identification of oral pathology with etiology, and treatment standards to warrant readiness of the MWD team. The financial investment in MWD programs should not only focus on the acquisition and training, but the appropriate management and care to maximize career longevity. Over two-thirds of tooth trauma had unknown etiology. Documenting the cause of TDI from handlers and veterinary personnel allows for assessment of susceptible MWDs and facilitates management to offset the risks. Mitigating and appropriately treating tooth trauma of the military working dog improves mission readiness of the combat team; directly safeguarding human lives around the world.

## Data availability statement

The raw data supporting the conclusions of this article will be made available by the authors, without undue reservation.

## Author contributions

KB: study conception and design, data collection, analysis and interpretation of results, and manuscript. SM: statistical analysis. TH: study conception, design, interpretation of results, and manuscript revision. All authors contributed to the article and approved the submitted version.
